# The ’bendy' basilar: progressive aneurysm tilting and arterial deformation can be a delayed outcome after coiling of large basilar apex aneurysms

**DOI:** 10.1136/neurintsurg-2018-013940

**Published:** 2018-05-17

**Authors:** Ansaar T Rai, Abdul R Tarabishy, SoHyun Boo, Jeffrey S Carpenter, Sanjay Bhattia

**Affiliations:** 1 Department of Interventional Neuroradiology, West Virginia University Hospital, Morgantown, West Virginia, USA; 2 Department of Neuroradiology, West Virginia University, Morgantown, West Virginia, USA; 3 Department of Neurosurgery, West Virginia University, Morgantown, West Virginia, USA

**Keywords:** aneurysm, coil, complication

## Abstract

**Background:**

Morphological changes in the basilar artery and the artery-aneurysm relationship following coiling of large basilar apex aneurysms may induce morbidity.

**Methods:**

The basilar artery radius-of-curvature was measured along its center line on volumetrically reconstructed images formatted along the plane of curvature. The aneurysm-tilt-angle was measured between the distal basilar and the vertical long axis of the aneurysm. The measurements were compared between small (<10 mm) and large (≥10 mm) aneurysms on baseline and follow-up studies. The volume (mm^3^) and mass (g) of the deployed coils was also compared.

**Results:**

Among 94 consecutive aneurysms, 62 (66%) were <10 mm and 32 (34%) were ≥10 mm. The mean aneurysm size and volume was 9 mm (±4) and 507 mm^3^(±1366) respectively. The median aneurysm follow-up was 24 months (IQR 6–59). There was no difference between the groups based on age, gender, or associated comorbidities. The coil mass was 0.4 g (±0.2) for aneurysms <10 mm and 1.9 g (±1.6) for aneurysms ≥10 mm (P<0.0001). The total coil volume was 32 (±20) mm^3^ for aneurysms <10 mm and 187 (±172) mm^3^ for aneurysms ≥10 mm (P<0.0001). Aneurysms ≥10 mm tilted 13.5^o^ (±14.4) compared with 1.1^o^ (±2.8) for aneurysms <10 mm (P<0.0001). The basilar artery became more curved by 1.3 (±9.4) mm for aneurysms ≥10 mm and 0.25 (±2.1) mm for aneurysms <10 mm (P=0.0002). Other than size of the coiled aneurysms no other factors correlated with the geometrical changes.

**Conclusion:**

Large coiled basilar apex aneurysms may be more prone to aneurysm tilting and bending of the basilar artery. Speculative causes include the weight of the coil mass and the biomechanical forces exerted on the coiled aneurysm.

## Introduction

A challenging microsurgical operation made basilar apex aneurysms one of the first to be treated with endovascular techniques.^1 2^ Over time, evolving skills and devices have made endovascular therapy the preferred method for treating most, if not all, basilar apex aneurysms. The mid- and long-term outcomes in terms of recurrence, retreatment, and re-bleeding favor this approach.^3 4^ Our observation of the tilting behavior of large coiled basilar apex aneurysms led us to undertake this study. This ‘complication’ of the effects of coiling on large basilar apex aneurysms and the basilar artery itself may lurk under the surface, possibly unrecognized and so potentially underreported. Some clues to the ‘bendy’ nature of the basilar artery and its neurological impact exist in the literature.^5 6^ Diseased arteries with underlying risk factors are more prone to this phenomenon^6^ and since arteries with aneurysms are by definition diseased, it is possible that in large aneurysms, the natural basilar bend, the flow dynamics, and the addition of a coil mass induces a structural change in the aneurysm-artery relationship which in extreme cases may cause significant morbidity. A recognition of this potential outcome in certain types of aneurysms may lead to modification of endovascular techniques and devices. The objective of this study was to determine if large basilar apex aneurysms (≥10 mm) are more prone to tilting after undergoing endovascular coiling procedures.

## Methods

The study was approved by the institutional review board. This is a retrospective analysis of basilar apex aneurysms treated with endovascular coiling, with or without adjunctive devices.

### Patient selection

A prospectively maintained aneurysm database was queried for patients undergoing endovascular treatment for basilar apex aneurysms. The recorded data included demographics and risk factors, ruptured versus unruptured status, total number of procedures per aneurysm, and type of aneurysm treatment, i.e., primary coiling versus stent or balloon assistance. The sample was divided into aneurysms with a maximum dimension of <10 mm or ≥10 mm and a retrospective analysis was performed as described next.

### Vascular analysis and measurements

**Figure 1:**
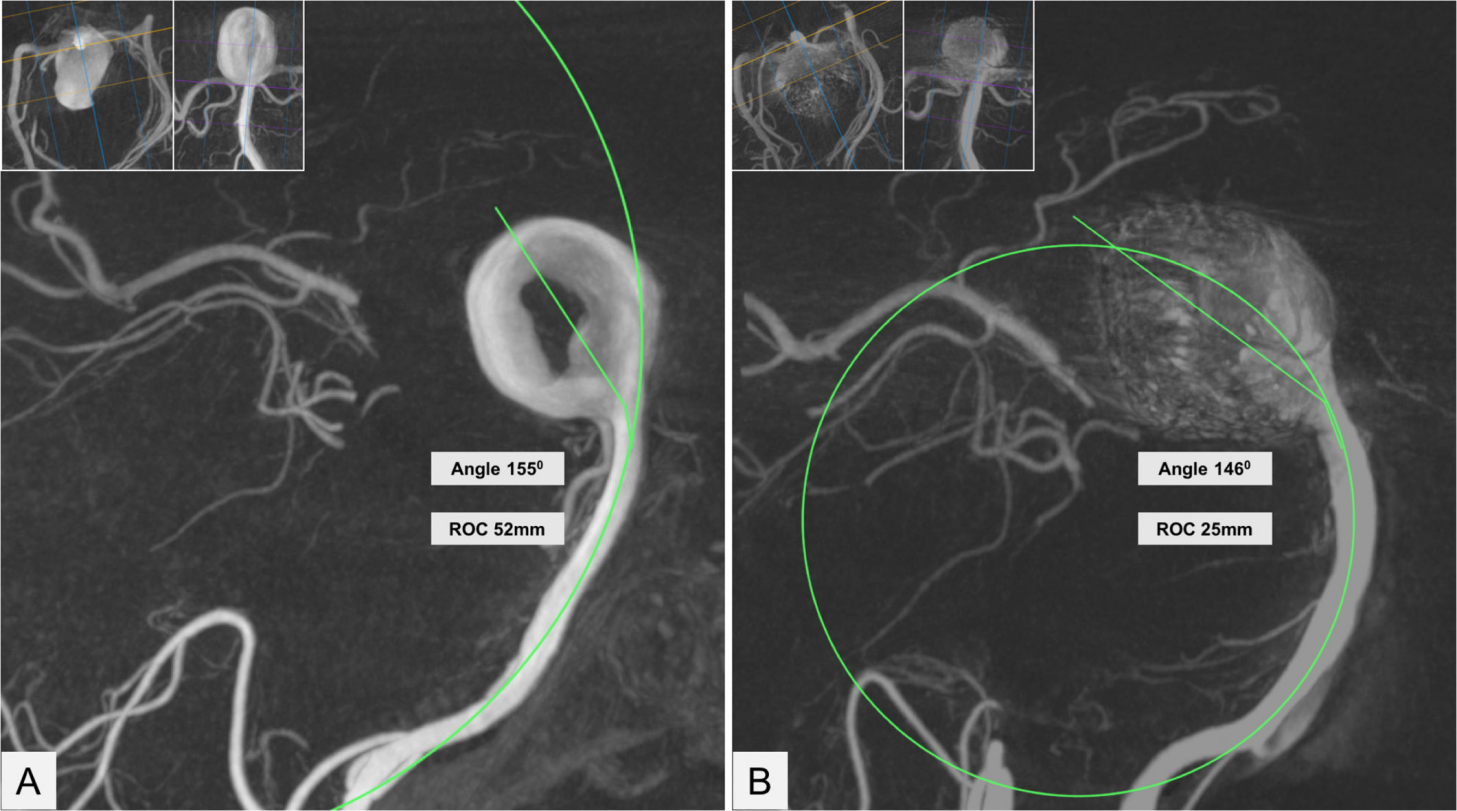
Methodology for measuring the radius of curvature and the aneurysm tilt angle. (A): Curved reformatted image generated from rotational angiography source data in the sagittal plane along the curvature of the basilar artery shows a large basilar apex aneurysm with central thrombus. The two inset images show curved reformats in the perpendicular axial and coronal planes utilized to isolate the curvature. The radius of curvature (ROC) is calculated from a circle superimposed on the center line of the basilar artery. The ROC is derived from the area of the circle. The aneurysm-tilt-angle is the angle between a line bisecting from the dome of the aneurysm to the aneurysm neck and a line from the aneurysm neck along the distal basilar artery center line. (B): Similarly generated curved reformatted image from a follow-up angiogram after Y-stenting and a total of three coiling procedures over 2 years shows reduction of the aneurysm-tilt-angle by 9 degrees and ROC by just over half. The total coil mass in the aneurysm was 6.4 grams.

The radius of curvature of the basilar artery and the aneurysm-tilt-angle were the two parameters that were measured on the baseline and the last follow-up studies. Source dicom data generated from rotational digital subtraction angiography (DSA) for the initial evaluation or DSA, CT angiography, and MR angiography for the follow-up studies was processed utilizing OsiriX MD software (Pixmeo, Bernex, Switzerland). Multiplanar curve reformatted images were generated with variable slab thicknesses to isolate the basilar artery along its curvature. The maximum radius of curvature was measured along the center line by placing a circle conforming to the basilar artery curvature and the radius was calculated from the area of the circle. For basilar artery with curvatures in two planes, for instance front-to-back and side-to-side, the curvature continuing along the long axis of the aneurysm was used as the primary measurement. Similarly, multiplanar curved reformats were used to define the artery-aneurysm junction. The aneurysm-tilt-angle was measured as the angle between a line bisecting the dome of the aneurysm to the aneurysm neck, and from the aneurysm neck to the distal 1/3rd of the basilar artery along its center line (figure 1). These measurements with the same frames of reference as the initial MPR reconstructed images were repeated on follow-up imaging. A smaller aneurysm-tilt-angle compared with the baseline angiogram indicated aneurysm tilting and a shorter radius of curvature compared with the baseline indicated a more curved basilar artery.

### Operative and device details

These included the total number of procedures per aneurysm, the use of adjunctive devices, the total number of coils deployed in each aneurysm, and the total length of coils (in centimeters) deployed. To account for the varying thickness of the coils, the total coil volume (mm^3^), and the packing density for each aneurysm was also calculated using commercial software (http://angiocalc.com/index.php). The coil volume for each coil is a function of the its length and primary outer diameter, the coil volume in turn impacts the packing density. The coil mass is the actual weight of all the implants. All other things being equal, a larger coil volume indicates a higher coil mass. However, the coil mass can be different for a given coil volume depending on the metal alloy used in the construction of the coil. Since, the effect of coil weight in larger aneurysms was speculated as a possible contributor to aneurysm tilting, we utilized proprietary vendor-supplied metrics for coil outer diameter (OD), coil length (cm), the number of wind, total wire length of the wind, the metal volume (cm^3^), and the density (g/cm^3^) of the platinum-tungsten alloy used for making the coil implant to calculate the mass of each coil (in grams) and the total mass of the conglomerate coil package (g) in the treated aneurysm. We also obtained the mass of the WEB intra-saccular device for different sizes (Microvention Terumo, Aliso Viejo, CA). The objective of this exercise was to provide a comparison of the intra-aneurysm implant mass for similar sized aneurysms treated with coils versus an intra-saccular device.

## Results

A total of 94 consecutive patients with basilar apex aneurysm treated with endovascular procedures were identified. Of these, 62 (66%) were <10 mm and 32 (34%) were ≥10 mm. The mean age was 55 (±10) years and there were 74 (79%) female patients. There was no difference between the two groups based on age, gender, or associated comorbidities. There were 51 (54%) unruptured and 43 (46%) ruptured aneurysms at initial presentation. There was no significant size difference between ruptured and unruptured aneurysms. The majority of patients, i.e., 73 (77.7%) underwent one procedure, 11 (11.7%) patients underwent two procedures, six (6.4%) patients underwent three procedures, and four (4.2%) patients underwent four procedures. Overall, coils only were used in 52 (55%) patients, adjunctive stent in 41 (44%) patients, and adjunctive balloon in one (1%) patient. For the initial procedure only, 66 (70.2%) patients were treated with primary coiling and 28 (29.8%) were treated with stent/coiling procedures. There was no significant difference in the rates of stent/coiling based on aneurysm size. The mean aneurysm size was 9 mm (±4) and the mean aneurysm volume was 507 mm^3^ (±1366). There was no difference in aneurysm size, the baseline radius of curvature, or the aneurysm angle based on the associated comorbidities (diabetes, hypertension, smoking, or hypercholesterolemia). The median aneurysm follow-up was 24 months (IQR 6–59).

The procedural differences and baseline geometric measurements are given for the entire cohort to include pre-operative information on native aneurysm-artery geometry and basilar artery radius of curvature (table 1). There was no significant difference in the baseline radius of curvature or the aneurysm-tilt-angle between the small and large aneurysms. As expected, aneurysms ≥10 mm had more total coils deployed with a higher total coil length and volume. There was no difference in the final packing density between the two groups, however significantly more coils were used in the larger aneurysms to achieve similar packing density as the smaller aneurysms. The coil mass in grams was calculated for select aneurysms (n=42) treated with one vendor coils for which technical specifications were available as discussed in the Methods section. The coil mass was 0.4 g (±0.2) for aneurysms <10 mm (n=26) and 1.9 g (±1.6) for aneurysms ≥10 mm (n=26), (P<0.0001).

**Table 1:** Baseline aneurysm and arterial geometry prior to endovascular procedure and implant details following the procedures is shown for small and large aneurysms

	Aneurysms <10 mm (n=62)	Aneurysms ≥10 mm (n=32)	P values
Baseline radius of curvature (mm)	39.2 (±16)	40.4 (±18)	0.9
Baseline aneurysm-tilt-angle (deg)	144 (±29)	146 (±29)	0.8
Total number of coils	6 (±3.5)	10 (±9)	0.0009
Total length of coils (cm)	48 (±31)	199 (±141)	<0.0001
Total coil volume (mm^3^)	32 (±20)	187 (±172)	<0.0001
Final packing density (%)	25 (±9)	21 (±8)	0.1

Table 2 shows the changes in radius of curvature and the aneurysm-tilt-angle as well as the change in these two parameters on the baseline and post-aneurysm coiling. The follow-up measurements for 69 patients in whom follow-up was available are given in table 2. The median follow-up was 24 months (IQR 6–59). The radius of curvature of the basilar artery and the aneurysm-tilt-angle significantly changed between aneurysms <10 mm versus aneurysms ≥10 mm from the baseline to the last follow-up study (table 2). Aneurysms ≥10 mm had a more curved basilar artery indicated by the shorter radius of curvature and the aneurysm-tilt-angle was significantly smaller, indicating a higher degree of posterior tilting. There was no significant association between underlying comorbidities such as hypertension, diabetes, hypercholesterolemia, and smoking with the geometric changes. In order to determine the effect of stent assistance in larger aneurysms we compared the change in radius of curvature and aneurysm-tilt-angle in aneurysms ≥10 mm (n=22) treated with or without stent assistance. There was no significant difference in these parameters based on addition of a stent and we did not measure immediate post-stent geometric changes as published previously.^7 8^ However, a difference could be missed because of the small sample size.

**Table 2:** Follow-up measurements for basilar artery radius of curvature and aneurysm-tilt-angle. The baseline measurements are from the initial preoperative angiography. The change in measurements is the difference between the last follow-up and the baseline preoperative angiography as calculated for each patient

	Aneurysms <10 mm (n=47)	Aneurysms ≥10 mm (n=22)	P values
Follow-up radius of curvature (mm)	36.5 (±14.5)	35.1 (±16)	0.6
Follow-up aneurysm-tilt-angle (deg)	146.3 (±29)	126.7 (±26)	0.001
Change in aneurysm-tilt-angle (deg)	1.1 (±2.8)	13.5 (±14.4)	<0.0001
Change in radius of curvature (mm)	0.25 (±2.1)	1.3 (±9.4)	0.0002

**Figure 2:**
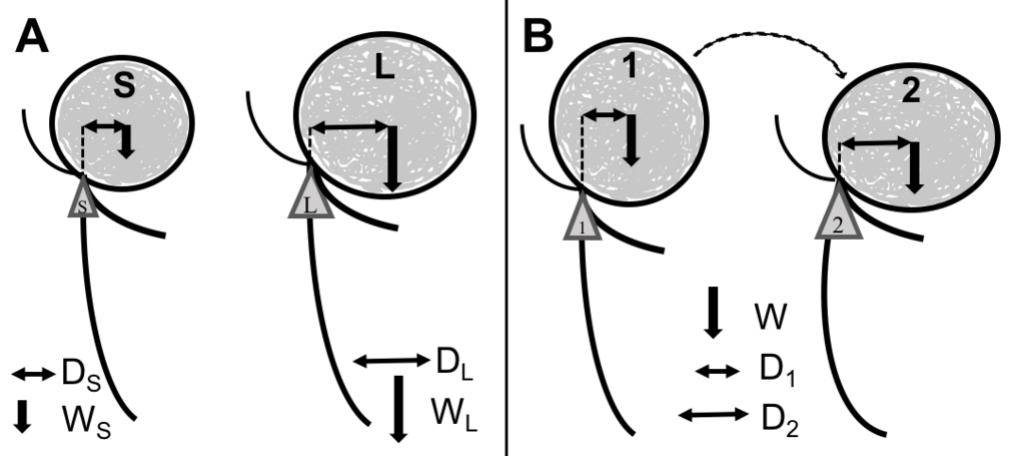
Bending forces (torque) applied at the neck in coiled aneurysms based on aneurysm size and tilt. (A): The larger aneurysm (L) not only has a higher coil mass (W_S_<W_L_) but the centroid of the coil mass is also further away (D_S_<D_L_) from the aneurysm base, resulting in a proportionately larger torque (T) represented by the triangles at the aneurysm neck, T=D x W. (B): Aneurysm tilting and basilar ‘bending’ may further increase the torque. As the large aneurysm tilts following coiling from position 1 to 2, the centroid shifts (D_1_<D_2_) further away from the neck. Thus, even without addition of more coils (W is the same for both aneurysms), the increased centroid distance can increase the torque at the aneurysm-artery junction potentially further, perpetuating the geometric change.

**Figure 3:**
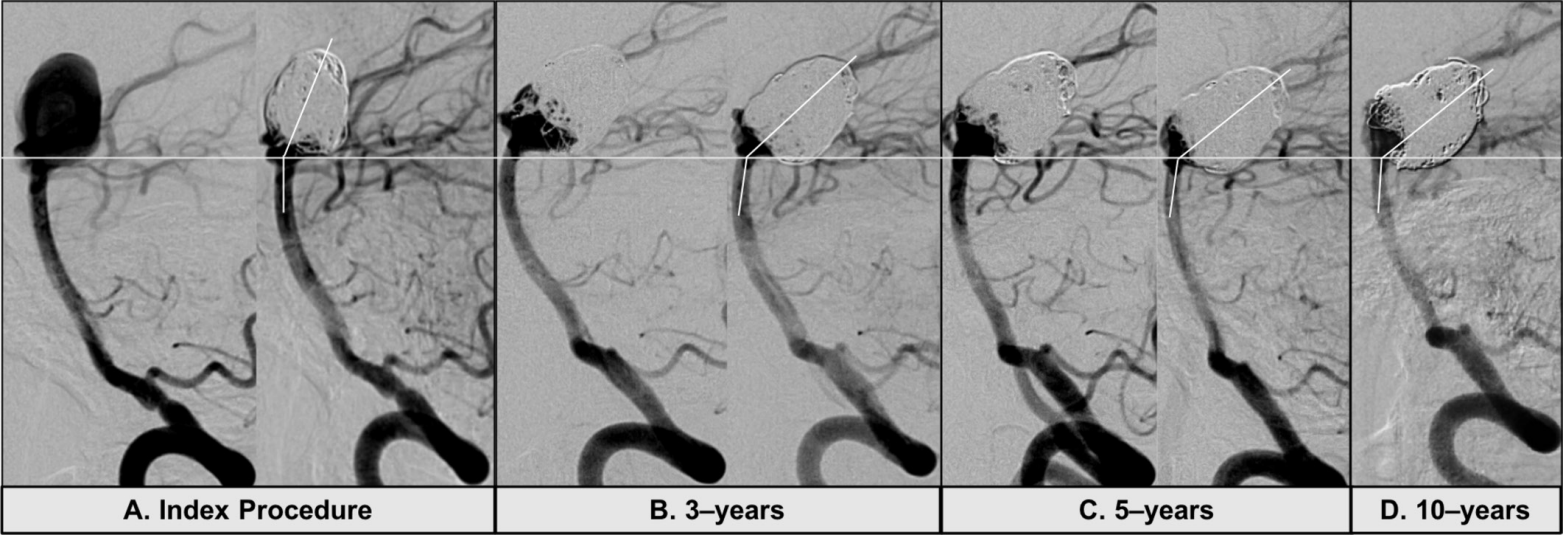
Example of gradual aneurysm tilting. (A):The index procedure shows immediately pre- and post-coiling images with good occlusion of the aneurysm. (B): The aneurysm was recoiled at 3 years due to recurrence at the neck. The radius of curvature (ROC) and the aneurysm-tilt-angle is smaller than the baseline procedure. (C): Additional coils were placed at 5 years due to slight recurrence at the neck. There is no change in the ROC but marginal decrease in the aneurysm-tilt-angle. (D): Follow-up angiography at 10 years from the index procedure shows slight recurrence at the neck but no change in the ROC or aneurysm-tilt-angle.

**Figure 4:**
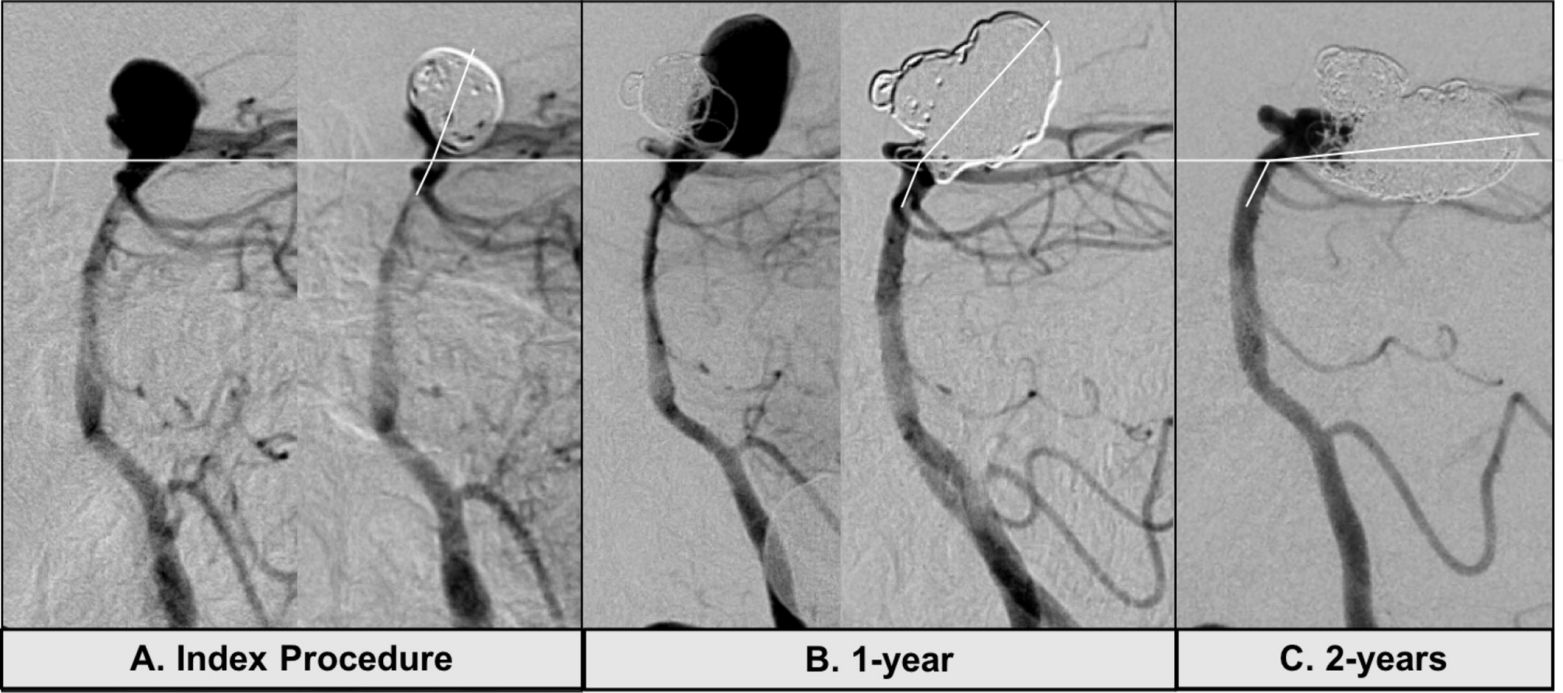
Example of extreme aneurysm tilting. (A): The index procedure shows immediately pre- and post-coiling images with good occlusion of the aneurysm. (B): There was a significant posterior recurrence at 1 year that was adequately coiled without stent assistance. (C): Follow-up angiogram 2 years after the initial procedure and 1 year after the second coiling shows minimal aneurysm recurrence at the neck but significant posterior tilting. Of note, no additional treatment was performed after the second procedure. The total coil mass in the aneurysm was at 4.2 grams.

**Figure 5:**
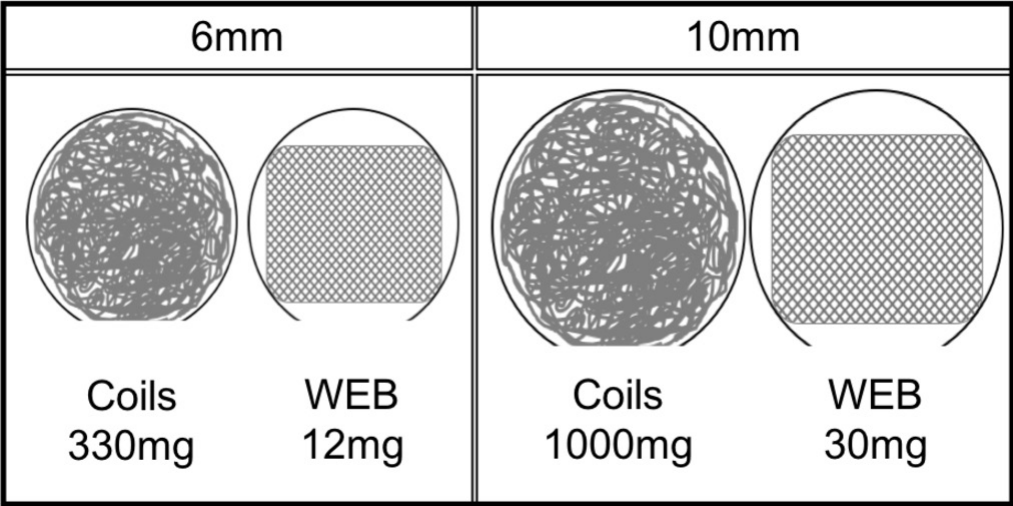
Comparison of intra-aneurysmal implant mass for a 6 mm and 10 mm aneurysm. The coil mass is based on the average coil mass in 6 mm aneurysms in the current data set. The mass of the WEB device is based on vendor-provided specifics for an 11×9 mm WEB device rated at 25–30 mg.

A possible effect of the coil mass and aneurysm size on the applied force and torque at the aneurysm artery junction and basilar curvature is presented in figure 2. Two case examples illustrate the progressive aneurysm tilting following coiling of large aneurysms. In the first case (figure 3), the change in tilt angle is observed over a 10-year period. In the second case (figure 4), more dramatic change is observed within a year and without additional treatment between the interval. The second case also resulted in significant morbidity due to the tilted aneurysm exerting mass effect on the mid brain and the floor of the third ventricle that ultimately required an endoscopic ventriculostomy. Lastly, for comparison the implant mass for coils versus the WEB (Microvention Terumo, Aliso Viejo, CA) intra-saccular device is shown for a 6 mm and a 10 mm aneurysm respectively (figure 5). The coil weight is based on the average mass of coils deployed in a 6 mm and a 10 mm aneurysm in our database and the mass of the small and large WEB devices is based on vendor specifications for a typical device recommended for treatment of aneurysm with these sizes.

## Discussion

A dime weighs 2.268 grams and a quarter 5.67 grams. The total coil mass in the first aneurysm (figure 1) at 6.4 grams is greater than the mass of a quarter and the coil mass in the third aneurysm (figure 5) at 4.2 grams almost equals two dimes. This mass is suspended at the end of a pliable ‘tube’, i.e., the basilar artery surrounded by fluid and subjected to constant pulsatile flow. This study was undertaken after observing significant morbidity in some patients following coiling of large basilar apex aneurysms that had tilted posteriorly, resulting in obstructive hydrocephalus. A similar case was reported by Sauvageau et al in 2004.^9^ Even without the addition of coils, giant basilar apex aneurysms can cause obstructive hydrocephalus^10–12^ and adequate treatment of these aneurysms remains an unmet need in endovascular neurosurgery. Our analysis shows that large aneurysms have a significantly higher tendency to undergo these changes as opposed to smaller aneurysms and hypothesizes that the addition of coils may induce a structural change in these large aneurysms. The posterior tilting is more pronounced than the change in radius of curvature of the basilar artery and it is possible that the tilting induces the change in curvature which, in turn, along with the added weight, causes further tilting.

One study analyzing the interaction between local hemodynamics and intra-aneurysmal flow in coiled basilar bifurcation aneurysms showed significant repetitive impingement on the coil mass due to the intra-aneurysmal flow.^13^ This water hammer effect may act as a force multiplier on the larger coil mass and the shifted centroid, contributing to aneurysm tilting (figure 2). Another recent study^14^ utilizing computational fluid dynamics (CFD) showed that recanalization of endovascularly treated basilar apex aneurysms was significantly associated with the proportion of intra-aneurysmal flow rate relative to the basilar artery flow rate (the intra-aneurysmal flow rate coefficient) and a higher coefficient was independently associated with higher recurrence rates. CFD analysis showed that patients with both posterior cerebral arteries (PCA) in a ‘hands-down’ configuration had almost all the blood from the basilar artery directed into the aneurysm^14^ and these patients had higher recurrence rates versus patients with a ‘hands-up’ configuration in whom the basilar flow was split three ways, with one component entering the aneurysm and two others going out of the PCAs. Thus, certain coiled aneurysms may be more prone to the water hammer effect of the pulsatile blood flow than others and theoretically in larger aneurysms with higher intra-aneurysm coil mass these biomechanical stresses may alter the vascular-aneurysm geometry. Additionally, associated vascular risk factors in these patients may play a role in changing the arterial morphology^6^ from the baseline,^15^ making the vasculature more susceptible to these interactions.

The addition of stents can alter the configuration of the PCAs in relation to the basilar apex, potentially narrowing the bifurcation angle.^7 8^ Whether this leads to reduced aneurysm recurrence is unproven but stent-assisted coiling does allow for a higher packing density^16^ that may indirectly reduce recurrences. Unilateral or bilateral PCA stenting in a ‘Y’ or kissing configuration by changing the vascular geometry may structurally support the distal basilar and the terminal branches acting as a counter anchor to the stress on the aneurysm caused by the coil mass and the higher torque at the neck in larger aneurysms (figure 2). The growth of the aneurysm around the coil mass may be asymmetric and, if posterior, will change the orientation with respect to the basilar artery. The adjacent brain parenchyma may have an effect on effecting the geometrical changes especially after the aneurysm has been repeatedly coiled and the interaction between a relative soft pulsatile mass changes to a firmer coiled mass. Additionally, older age and diseases that affect the vascular morphology^5 17–19^ such as hypertension, diabetes, and smoking may impact or exaggerate these geometric changes in larger aneurysms. Our second patient (figure 5) had a large posterior recurrence following the initial coiling and was retreated. However, there was no aneurysm recurrence and no additional treatment between the repeat coiling and the follow-up angiogram 1 year later which still showed dramatic posterior tilting without aneurysm recurrence. This suggests that the large coil mass (4.2 g) combined with the water hammer effect from the pulsatile blood flow potentially induced this structural change. Another possibility is that the dense coil mass, made of predominantly radio-opaque platinum alloys, was obscuring any intra-aneurysmal flow on the digital subtraction angiography, which could have also contributed to the posterior tilt. Newer braided coils^20^ and intrasaccluar flow disruptive devices^21^ constructed with more radiolucent alloys and less platinum may allow for better visualization of intra-aneurysmal contrast flow and perhaps a truer estimation of aneurysm occlusion.

Flow diversion for large basilar apex aneurysms has been reported^22 23^ with mixed technical and safety results. The use of intra-luminal as opposed to intra-aneurysmal devices, is appealing because of eliminating or reducing the coil mass especially within large aneurysms. In the same vein, intra-saccular devices that act by disrupting blood flow such as the WEB device (Microvention Terumo, Aliso Viejo, CA) by virtue of having significantly lower mass (figure 3), may theoretically prevent these morphological outcomes. But currently for aneurysms larger than 12 mm these devices do not exist apart from the fact that these assumptions are speculative and not supported by data. A recent publication has demonstrated higher rates of permanent occlusion with microsurgical clipping of basilar apex aneurysms than endovascular therapy although with a higher rate of cranial nerve deficits and hemiparesis.^24^ Thus, the benefits of a higher earlier occlusion may not outweigh the surgical complication rate and the lower morbidity induced by tilted aneurysms.

### Limitations

This is primarily an observational study showing the propensity of larger coiled aneurysms to undergo a geometric change with reference to the basilar artery. Other than aneurysm size, the study does not offer any tangible causes for this phenomenon and all the potential causes discussed are speculative. An aneurysm size of 10 mm was arbitrarily used to dichotomize aneurysms into large and small and, given the wide variation in the degree of geometric change, it was not possible to do a receiver operator curve analysis to identify a size after which an aneurysm ‘tilts’ or changes the radius of curvature of the basilar artery. Moreover, we observed that in some aneurysms these changes were more abrupt and dramatic, while in others these were less prominent and more gradual, thus not allowing any meaningful conclusions to be drawn with reference to the time dependency of these changes. Similarly, while we speculate that adjunctive stent placement may help basilar-PCA transition and ‘anchor’ the aneurysm neck, our data did not show any correlation with stenting. It is possible that such a relationship exists but could not be demonstrated because of our sample size.

## Conclusions

Large, recurring basilar apex aneurysms continue to present a therapeutic challenge for endovascular therapy. Multiple procedures may be needed to achieve stable angiographic occlusion but, in the process, may induce a structural change in the aneurysm geometry and the basilar artery. Accumulation of a heavy coil mass in the process, coupled with biomechanical stresses, may contribute to this phenomenon. Possible, but speculative, strategies to reduce these geometric changes may include structural support using adjunctive stents, intraluminal flow diversion, intrasaccluar flow disruptive devices, or coils with lighter mass and better radiolucency.
